# Evaluation of short synthetic antimicrobial peptides for treatment of drug-resistant and intracellular *Staphylococcus aureus*

**DOI:** 10.1038/srep29707

**Published:** 2016-07-11

**Authors:** Mohamed F. Mohamed, Ahmed Abdelkhalek, Mohamed N. Seleem

**Affiliations:** 1 Department of Comparative Pathobiology, College of Veterinary Medicine, Purdue University, West Lafayette, IN 47907, USA; 2 Purdue Institute for Inflammation, Immunology, and Infectious Disease, Purdue University, West Lafayette, IN 47907, USA

## Abstract

Methicillin-resistant *Staphylococcus aureus* (MRSA) infections present a serious challenge because of the emergence of resistance to numerous conventional antibiotics. Due to their unique mode of action, antimicrobial peptides are novel alternatives to traditional antibiotics for tackling the issue of bacterial multidrug resistance. Herein, we investigated the antibacterial activity of two short novel peptides (WR12, a 12 residue peptide composed exclusively of arginine and tryptophan, and D-IK8, an eight residue β-sheet peptide) against multidrug resistant staphylococci. *In vitro*, both peptides exhibited good antibacterial activity against MRSA, vancomycin-resistant *S. aureus*, linezolid-resistant *S. aureus*, and methicillin-resistant *S. epidermidis*. WR12 and D-IK8 were able to eradicate persisters, MRSA in stationary growth phase, and showed significant clearance of intracellular MRSA in comparison to both vancomycin and linezolid. *In vivo*, topical WR12 and D-IK8 significantly reduced both the bacterial load and the levels of the pro-inflammatory cytokines including tumor necrosis factor-α (TNF-α) and interleukin-6 (IL-6) in MRSA-infected skin lesions. Moreover, both peptides disrupted established *in vitro* biofilms of *S. aureus* and *S. epidermidis* significantly more so than traditional antimicrobials tested. Taken together, these results support the potential of WR12 and D-IK8 to be used as a topical antimicrobial agent for the treatment of staphylococcal skin infections.

The rapid development and spread of bacterial resistance to conventional antibiotics, particularly those associated with staphylococcal infections, has become a serious global concern. Nearly 11,000 people die each year from a methicillin-resistant *Staphylococcus aureus* (MRSA)-related infection alone in the United States; this figure represents nearly half of all fatalities caused by antibiotic-resistant bacteria pathogens^1^,^2^. *Staphylococcus aureus* is the pathogen most frequently isolated from human skin and wound infections^2^. Staphylococcal biofilms and toxins can evade the host immune system, leading to recurring/chronic infections, prolonging inflammation, and hindering the process of wound healing^3^. Furthermore, the emergence of MRSA strains exhibiting resistance to topical drugs of choice, including mupirocin and fusidic acid, is a significant public health challenge that requires novel therapeutic alternatives^4^. Antimicrobial peptides (AMPs) have shown significant promise in recent years as novel therapeutic agents to treat infections caused by multidrug-resistant pathogens^1^. AMPs are a major component of the human skin’s innate immunity and a decrease in the production of AMPs in the dermis is associated with increased susceptibility to skin infection with *S. aureus* in humans^5^. In addition to possessing potent antibacterial activity, AMPs have several unique advantages over traditional antibiotics. These advantages include AMPs possess a broad spectrum of activity, low potential for resistance development, ability to neutralize virulence factors released by pathogens, and ability to modulate the host immune response^1^. However, there are several limitations for utilizing naturally-derived AMPs, particularly for treatment of invasive infections. These limitations include host toxicity, degradation by proteases, extensive serum binding, loss of antimicrobial activity in the presence of physiological concentration of salts, and high cost of production due to their complex design. These limitations need to be addressed, and new avenues need to be pursued in order to transform AMPs into novel therapeutic agents capable of being used clinically. One avenue gaining momentum is the utilization of AMPs as topical antibacterial agents. Multiple AMPs have already reached various stages of clinical trials for the treatment and prevention of bacterial infections^1^. In the present study, the *in vitro* and *in vivo* antibacterial activity of two unique AMPs, WR12 and IK8 all D (D-IK8), was investigated against multidrug-resistant *S. aureus*. These two peptides have several advantages, including potent antibacterial activity, high selectivity, and short and simple sequences (8–12 amino acids). Their simplified sequences should facilitate their rapid production, decrease their cost of synthesis, and accelerate their translational clinical applications^6^,^7^. WR12 (RWWRWWRRWWRR) is a *de novo* designed short peptide composed of 12 amino acids with broad-spectrum antibacterial activity. It is composed exclusively of arginine and tryptophan and is designed to form standard amphipathic helices with six cationic charges and 50% hydrophobicity^6^. Peptide D-IK8 (irikirik) is a short synthetic β-sheet forming peptide composed only of eight amino acids with four cationic charges and 50% hydrophobicity. Importantly, substitution of the L-amino acids of IK8 with the D-isoform provides resistance to enzymatic degradation by animal and bacterial proteases^7^. Both WR12 and D-IK8 kill bacteria by disrupting bacterial membranes, leading to leakage of intracellular contents and, consequently, bacterial death. This mechanism explains the low emergence of bacterial resistance observed after serial passage with sub-inhibitory concentrations of peptides^6^,^7^.

The aim of this study was to investigate the spectrum of antibacterial activity of the designed peptides against a collection of important multidrug-resistant strains of staphylococci isolated from clinical settings, to assess their ability to kill persisters and MRSA in stationary phase of growth, to explore their antibiofilm activity and their ability to clear intracellular infections, to investigate their ability to be used in combination with conventional antibiotics, and to assess their efficacy and immune-modulatory effect in a murine model of MRSA skin infection.

## Results and Discussion

### Antimicrobial activity

We explored the antibacterial activity of WR12 and D-IK8 against multidrug-resistant strains of staphylococci, including methicillin-sensitive *S. aureus* (MSSA), MRSA, vancomycin-intermediate *S. aureus* (VISA), vancomycin-resistant *S. aureus* (VRSA), linezolid-resistant *S. aureus* and methicillin-resistant *S. epidermidis* (MRSE) (Tables 1 and 2). WR12 exhibited strong antibacterial activity against MRSA, inhibiting 50% of the strains (MIC_50_) at a concentration of 2 μM and inhibiting 90% of the strains (MIC_90_) at a concentration of 8 μM. The MIC_50_ and MIC_90_ of D-IK8 against MRSA strains were found to be 8 and 16 μM, respectively. Moreover, WR12 and D-IK8, demonstrated good activity against multiple clinical isolates of MRSA, particularly MRSA USA300, a community-associated strain responsible for major outbreaks of staphylococcal skin and soft-tissue infections (SSTI)^8^. Similarly, potent antibacterial activity of these peptides was observed against other important clinical MRSA isolates (USA500, USA200, and USA100) that exhibit resistance to various antibiotic classes, including fluoroquinolones, macrolides, lincosamides, and aminoglycosides.

WR12 and D-IK8 also showed potent antibacterial activity against all tested VRSA isolates that were resistant to vancomycin and teicoplanin, with MIC values ranging from 4–8 and 8–16 μM, respectively (Table 2). It is important to note that 85% of the VRSA strains we examined were isolated from wounds; thus the studied AMPs have potential to be used as a topical antimicrobial agent for the treatment of multidrug-resistant staphylococcal skin and wound infections^9^.

The superior antibacterial activity of WR12 over D-IK8 may be attributed to the higher cationic charge and the increased length of amino acids of WR12 (12 residues) compared to D-IK8 (8 residues) (Supplementary Table S2). Indeed, WR12 permeabilized the staphylococcal membrane more potently than D-IK8. As demonstrated in Supplementary Figure S1, at 5 × MIC, WR12 and D-IK8 caused more than 95% and 76% leakage of preloaded calcein dye within 60 minutes, respectively.

In contrast to WR12 and D-IK8, we detected very low activity and high level of resistance in staphylococci strains against the natural derived human AMP cathelicidin (LL-37) that protects human skin from bacterial infections^10^. LL-37 showed activity only against two strains of staphylococci (*S. aureus* ATCC 6538 and *S. epidermidis* ATCC 35984 “NRS 101”) with a MIC value equal to 16 μM. The MIC values of LL-37 against all other strains were more than 128 μM. Our results correlate with previous reports that have found high levels of resistance to LL-37 in clinical MRSA strains compared with MSSA bacterial isolates^11^. The emergence of resistance among clinical isolates to natural peptides, such as LL-37, could be one of the factors that has contributed to their global epidemic^12^.

We evaluated the activity of pexiganan against four isolates of staphylococci represent different antibiotic susceptibility phenotypes: MSSA, MRSA, MRSE, and VRSA (Supplementary Table S3). Pexiganan is an analog of the magainin peptides isolated from the skin of the African clawed frog. Pexiganan is the first AMPs that has advanced furthest in clinical trials for the treatment of diabetic foot ulcers. Pexiganan demonstrated comparable activity to D-IK8 and less activity than WR12 against MSSA, MRSA and VRSA (MIC of pexiganan is 16, 16, 32 μM against MSSA, MRSA and VRSA, respectively). However, pexiganan demonstrated enhanced activity than WR12 and D-IK8 against MRSE (MIC, 1 μM) (Supplementary Table S3). Ge *et al*. reported that the MIC_50_ and MIC_90_ of pexiganan against large panel of *S. aureus* isolates were 8 and 16 μg/ml, respectively^13^. A more recent study reported that pexiganan MIC values among MSSA and MRSA strains isolated from diabetic foot infections ranged from 16 to 32 μg/ml^14^. It is important to note that pexiganan have 22-amino-acid residues in contrast to WR12 (12 residues) and D-IK8 (8 residues). This ensures a relatively lower cost of production of WR12 and D-IK8 compared to pexiganan.

### Antimicrobial activity in physiological concentrations of salts

One major limitation with the clinical translation of AMPs is their potential inactivation by salts present in the human body. Therefore, we tested the MIC of peptides against MRSA USA300 in cation-adjusted Mueller Hinton medium and in regular MHB, for comparison (Supplementary Table S4). We did not find a significant difference in the MIC value of the peptides in the two conditions (there is no difference in the MIC of WR12 and only one fold increase of D-IK8 and pexiganan in the presence of cation-adjusted Mueller Hinton medium). To ensure that these peptides remain active in the presence of higher concentrations of cations, we tested the peptides in higher concentration of salts. As demonstrated in Supplementary Table 2, there was no difference in the MIC of WR12 in the presence of 150 mM NaCl. However, there was a two-fold increase in the MIC observed for D-IK8 and pexiganan at the same conditions. In the presence of a physiological concentration of MgCl_2_ (2 mM), we observed a one-fold increase in the MIC of WR12 and a two-fold increase in the MIC of D-IK8 or no change in the MIC of pexiganan, compared to regular MHB (Supplementary Table S4). The superior salt stability of WR12 compared to D-IK8 and pexiganan is attributed to its amino acid composition. WR12 is composed of tryptophan and arginine residues which are known to improve antimicrobial activity under challenging salt conditions^6^,^15^,^16^. In contrast, many well-studied AMPs (such as LL-37, human β-defensin-1, gramicidins, bactenecins, and magainins) demonstrated substantially reduced antibacterial activities under the same conditions^17^. Previously, Turner *et al*. reported that LL-37 and HNP-1 demonstrated a 12-fold and 100-fold increase in the MIC of MRSA, respectively, when 100 mM NaCl was added to the test medium^18^. The ability to resist the effects of salt provide a selective advantage for WR12 peptide for potential therapeutics in physiological solutions.

### Bacterial killing kinetics

**Figure 1:**
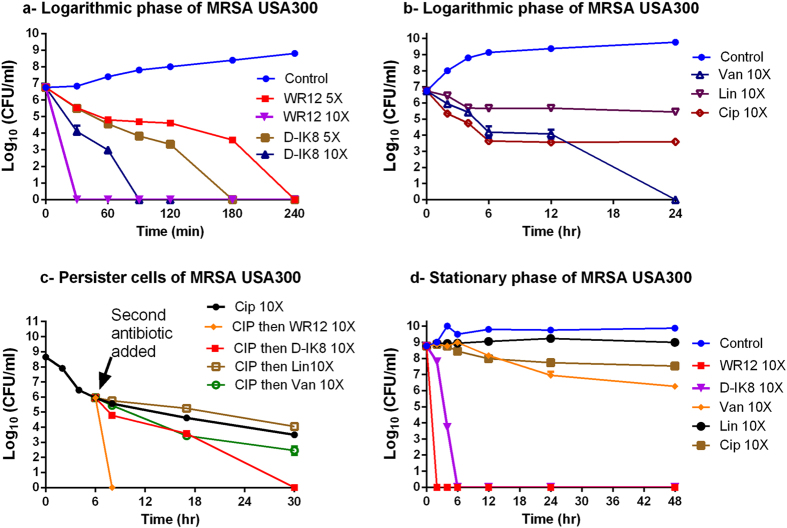
The kinetics of killing of peptides and antibiotics against logarithmic, persister cells and stationary phase of MRSA USA300. (**a**) Logarithmic phase of MRSA USA300 exposed to peptides (WR12, D-IK8) at 5X and 10X MIC or (**b**) antibiotics at 10X MIC. (**c**) The kinetics of killing of persister cells and (**d**) stationary phase of MRSA USA300 exposed to peptides (WR12, D-IK8) and antibiotics at 10X MIC. In Fig. 1c, CIP means treatment of MRSA with ciprofloxacin at 10X MIC for 6 hr then the surviving persisters were exposed to peptides or antibiotics at 10X MIC as the arrow pointed. Untreated samples served as a control. Abbreviations, Van, vancomycin; Lin, linezolid; Cip, ciprofloxacin. The results are given as means ± SD (no = 3); data without error bars indicate that the SD is too small to be seen.

After confirming that the AMPs possessed excellent antimicrobial activity against multidrug-resistant staphylococcal clinical isolates, we next assessed the killing kinetics of these AMPs. Both peptides showed concentration-dependent killing of MRSA USA300. WR12 showed fast bactericidal activity and was capable of completely eliminating a high starting inoculum of MRSA USA300 (5.6 × 10^6^ CFU/ml) within 30 minutes and 240 minutes at 10 × and 5 × MIC, respectively (Fig. 1a). D-IK8 showed slower bactericidal activity with complete clearance of MRSA within 90 minutes and 180 minutes at 10 × MIC and 5 × MIC, respectively. The rapid bactericidal activity of WR12, when compared to D-IK8, is mainly attributed to its ability to more rapidly permeabilize the staphylococcal membrane. In contrast to AMPs, conventional antibiotics demonstrated slower killing kinetics at 10 × MIC. As shown in Fig. 1b, vancomycin only produced a 2.5-log reduction after 12 hours of exposure and required 24 hours to completely eliminate MRSA USA300. It is worth noting that frequent clinical failure in MRSA patients receiving vancomycin treatment has been linked to the poor bactericidal activity of this drug^19^. Ciprofloxacin showed bactericidal activity by reducing the starting inoculum of MRSA USA300 3-log within six hours; however, complete clearance was not achieved, even after 24 hours of exposure. Linezolid, as expected, showed a bacteriostatic effect reducing MRSA USA300 starting inoculum by 1.3-log after 24 hours of treatment. AMPs with fast bactericidal activity have several advantages over their counterparts, conventional antibiotics, including limiting spread of infection, improving outcome of the disease, reducing the potential emergence of bacterial resistance, and reducing duration of treatment^20^.

### Efficacy of peptides on persister cells and stationary phases of MRSA

Persister cells are phenotypic variants of the normal bacterial population. They are extremely tolerant to antimicrobials and contribute to chronic and latent infections^21^,^22^,^23^,^24^. To assess the ability of AMPs to eradicate persister cells, MRSA was treated with ciprofloxacin in order to produce persister cells. When treated with ciprofloxacin, MRSA USA300 (in exponential growth phase) produces a biphasic killing pattern that results in surviving persister cells (Fig. 1c). The subsequent addition of conventional antimicrobials such as vancomycin or linezolid had minimal impact in reducing the number of persisters, which is in agreement with previous studies^22^,^25^,^26^. However, treatment with WR12 and D-IK8 resulted in complete eradication of persister cells after 2 and 24 hours, respectively (Fig. 1c).

The ability of both WR12 and D-IK8 to kill MRSA persisters led us to next assess their impact on stationary-phase *S. aureus* which is known to be tolerant to many antimicrobial agents^22^,^25^,^26^. Treatment of stationary-phase MRSA USA300 with WR12 and D-IK8 resulted in complete eradication of MRSA after 2 and 6 hours, respectively (Fig. 1d). With the exception of vancomycin, conventional antibiotics (ciprofloxacin and linezolid) did not have any effect on stationary-phase MRSA. Vancomycin had minimal impact in reducing the number of stationary-phase MRSA by only 2.4-log after 48 hours of exposure. The superior activity of our AMPs, when compared to conventional antibiotics, against persisters and stationary phase MRSA could be explained by their unique antimicrobial mechanism of action. Many antibiotics require growing and metabolically active bacteria to exert their antimicrobial effects and inhibit intracellular bacterial targets including nucleic acids (ciprofloxacin) or protein (linezolid) or cell wall synthesis (vancomycin)^27^. In contrast, the positive charge present in WR12 and D-IK8 serves as a point of attraction with cell membranes and consequently targeted-disruption of bacterial membrane and leakage of intracellular contents^6^,^7^. This unique mechanism of action of AMPs does not require cells to be metabolically active and is not impaired by the dormant and quiescent state of bacteria (stationary and persister cells)^27^. The results garnered lend valuable insight into using AMPs as a possible future therapeutic option for the treatment of persistent bacterial infections.

### Efficacy of peptides on *Staphylococcus* biofilms

**Figure 2:**
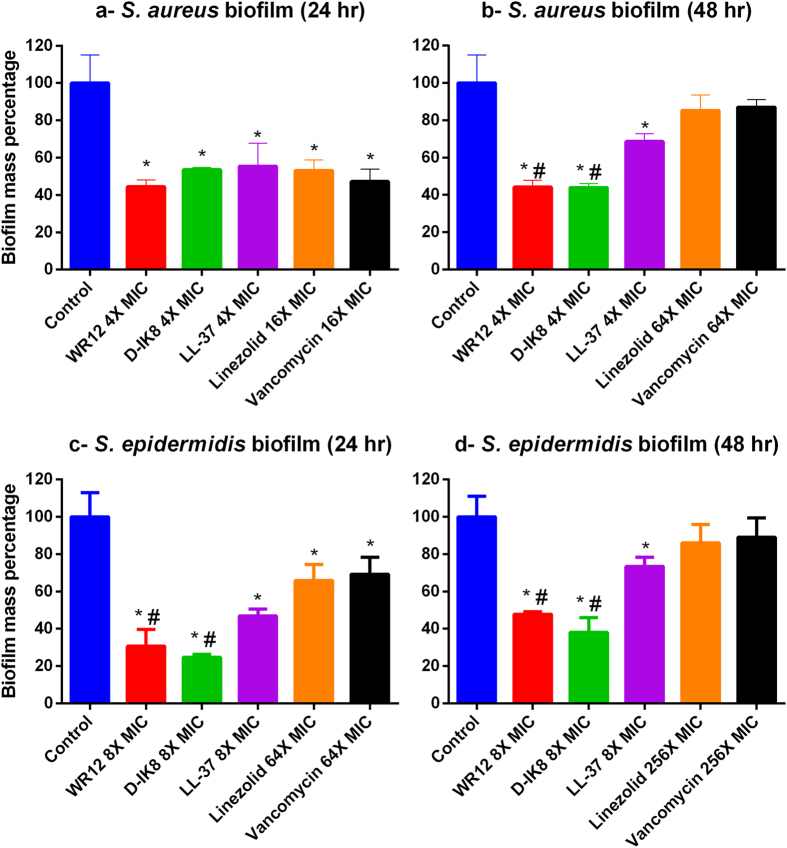
The effect of peptides (WR12, 0and LL-37) and antibiotics (vancomycin & linezolid) on 24 hr (**a,c**) and 48 hr (**b,d**) old biofilms of *S. aureus* (**a,b**) and *S. epidermidis* (**c,d**). The adherent biofilm stained by crystal violet, then the dye was extracted with ethanol, measured at 595 or 490 nm absorbance and presented as percentage of biofilm reduction compared to untreated wells “control”. All experiments were done in triplicate for statistical significance. The two tailed Student *t* test, was used to determine statistical significance between two groups. One asterisk (*) indicates statistically different than control (*p* < 0.05). Symbol (#) indicates statistically different than the antibiotic treated wells (*p* < 0.05). Detailed *P* values are listed: (**a**): WR12 vs control, 0.0032; D-IK8 vs control, 0.0058; LL-37 vs control, 0.0166; linezolid vs control, 0.0072; vancomycin vs control, 0.0047. (**b**) WR12 vs control, 0.0033; D-IK8 vs control, 0.0031; LL-37 vs control, 0.0251; linezolid vs control, 0.2122; vancomycin vs control, 0.2284; WR12 vs LL-37, 0.0015; WR12 vs linezolid, 0.0015; WR12 vs vancomycin, 0.0014; D-IK8 vs LL-37, 0.0007; D-IK8 vs linezolid, 0.0015; D-IK8 vs vancomycin, 0.0011. (**c**) WR12 vs control, 0.0016; D-IK8 vs control, 0.0006; LL-37 vs control, 0.0025; linezolid vs control; 0.0194 vancomycin vs control, 0.0283; WR12 vs LL-37, 0.0466; WR12 vs linezolid, 0.0079; WR12 vs vancomycin, 0.0064; IK8-D vs LL-37, 0.0008; D-IK8 vs linezolid, 0.0079; D-IK8 vs vancomycin, 0.0011. (**d**) WR12 vs control, 0.0012; D-IK8 vs control, 0.0014; LL-37 vs control, 0.0188; linezolid vs control, 0.1763; vancomycin vs control, 0.2786; WR12 vs LL-37, 0.0009; WR12 vs linezolid, 0.0026;WR12 vs vancomycin, 0.0024; D-IK8 vs LL-37, 0.0027; D-IK8 vs linezolid, 0.0026; D-IK8 vs vancomycin, 0.0025.

Biofilm formation is one of the major virulence factors of *S. aureus*^28^. The polysaccharide matrix of biofilm protects bacteria from host immune defenses and hinders the ability of antibiotics to target deep-seated bacteria residing within the biofilm^29^. Furthermore, biofilms act as an infectious niche with sustained release of bacteria inside the host, which leads to chronic infection, relapses, life-threatening bloodstream infections, and treatment failure^28^. Given the serious challenges associated with staphylococcal biofilms and their role in promoting recurring infections in the host, we next moved to assess whether our peptides are capable of disrupting mature biofilms (formed after 24 and 48 hours) of both *S. aureus* and *S. epidermidis*. As shown in Fig. 2a, both peptides (at 4 × MIC) significantly disrupted the 24 hour mature biofilms of *S. aureus*, reducing biofilm mass by 50%. Vancomycin and linezolid were required at a higher concentration (16 × MIC) to reduce the same percentage of biofilm mass (*p* < 0.05) (Fig. 2a). Although some conventional antibiotics might be capable of disrupting 24 hour-mature bacterial biofilm, most antibiotics are not effective against 48 hour-mature biofilms due to the dormant state of growth of the bacterial cells present within the mature-biofilms^30^. To examine whether the potential therapeutic application of WR12 and DIK-8 could be expanded beyond just inhibition of 24 hour biofilm, the ability of both peptides to disrupt 48 hours-mature staphylococcal biofilm was tested. As expected, we observed that the mature 48 hour *S. aureus* biofilms were resistant to antibiotics with no significant reduction in the biofilm mass observed, even at very high concentrations (64 × MIC) (Fig. 2b). Interestingly, WR12 and DIK-8 (at 4 × MIC) showed more than 50% reduction of biofilm mass (*p* < 0.05) (Fig. 2b).

Next, we evaluated the ability of our peptides to disrupt established biofilms of *S. epidermidis* ATCC 35984 (NRS 101), a clinical high slime producer strain isolated from septicemic patients with a colonized intravascular catheter^31^. This strain is a multidrug-resistant strain, exhibiting resistance to methicillin, erythromycin, kanamycin, gentamicin, clindamycin, and trimethoprim. The great thickness of the slime matrix of *S. epidermidis* biofilms makes it extremely resilient to antibiotic penetration^32^. Hence, the 24 hour-mature *S. epidermidis* biofilms were less susceptible to vancomycin and linezolid, even at 64 × MIC, showing only 30–35% biofilm inhibition. The 48 hour-mature biofilms were not susceptible to the effect of both antibiotics action, even at a very high concentration (256 × MIC). WR12 and DIK-8 (at 8 × MIC) significantly reduced biofilm mass by more than 70% and 50% in 24 and 48 hour-mature biofilms, respectively (*p* < 0.05) (Fig. 2c,d). The studied peptides proved to be far superior to antibiotics against biofilm due to their amphipathic nature and their high cationic charge, which may facilitate their penetration through the extracellular biofilm matrix. Furthermore, we included the known biofilm inhibitor, LL-37, as a control^33^. In our studies, LL-37 disrupted both biofilms of staphylococci regardless of the maturation stage of the biofilm. Interestingly, WR12 and D-IK8 showed improved activity compared to LL-37, particularly in 48 hour mature biofilms of both species of staphylococci (Fig. 2). This may be related to the more potent antimicrobial activity and amphipathicity of the designed peptides compared to LL-37.

### Combination therapy analysis

The potent antimicrobial activity of the AMPs indicated that they have the potential to be used alone for treatment of skin infections caused by *S. aureus*. Although the use of a single agent to treat skin infections caused by *S. aureus* is the most commonly used practice in clinical settings, combination therapy has several advantages. These advantages include minimizing the likelihood of emergence of bacterial drug-resistance, lowering the doses required for each antibacterial agent thus mitigating drug toxicity, and expanding the spectrum of pathogens that can be targeted^34^. Furthermore, several topical treatments currently used for treatment of skin infections involve a combination of more than one antibiotic, such as Polysporin (bacitracin, polymyxin B sulfate, and gramicidin) and Neosporin (bacitracin, neomycin, and polymyxin B sulfate)^35^. Thus, identifying AMPs to pair with conventional antibiotics used for treatment of *S. aureus* infections has good potential to expand available treatment options. Keeping the above points in mind, we were curious to assess the synergistic action of WR12 and D-IK8 in combination with each other and with conventional antibiotics against four staphylococcal isolates. The isolates were chosen to represent different antibiotic susceptibility phenotypes: MSSA, MRSA, MRSE, and VRSA.

As presented in Table 3, WR12 and D-IK8 displayed synergistic activity when combined against MSSA, MRSA, and MRSE but not against VRSA. The FIC indices against all isolates except VRSA varied from 0.27 to 0.38. When our peptides were combined with antibiotics, WR12 proved to be superior to D-IK8, as it displayed potent synergism with most topical antibiotics (fusidic acid and mupirocin) and systemic antibiotics (daptomycin, teicoplanin, vancomycin, linezolid, ciprofloxacin, meropenem and oxacillin) against most tested strains with FIC indices ranging from 0.26 to 0.5. D-IK8 demonstrated synergism with fusidic acid and daptomycin in all four strains tested with FIC indices ranging from 0.26 to 0.5. D-IK8 also showed synergism with teicoplanin in two strains and with oxacillin, vancomycin, meropenem, and linezolid in one strain.

Remarkably, we found that both WR12 and D-IK8 showed potent synergism with vancomycin against a VRSA isolate (with a low FIC index of 0.27). Since vancomycin is considered to have nephrotoxicity at higher concentrations, decreasing the therapeutic doses required to treat staphylococcal infection is beneficial, as it will reduce the adverse side effects in affected patients^34^. Notably, there were no antagonistic interactions recorded between the peptides and antimicrobials. The synergistic interaction between peptides and antibiotics could be a result of the membrane permeabilization action of peptides, leading to more penetration of antibiotics inside bacterial cells and augmented killing^13^,^14^. The potent and broad synergism observed for WR12 compared to D-IK8 is attributed to its rapid and more potent membrane permeabilization (Supplementary Figure 1).

### Re-sensitization of VRSA to vancomycin, teicoplanin, and oxacillin in the presence of a sub-inhibitory concentration of peptides

As a strong synergistic relationship was observed with the peptides and vancomycin against the VRSA10 strain, we hypothesized that these peptides could be used to re-sensitize vancomycin and teicoplanin-resistant *S. aureus* (VRSA) strains to the effect of vancomycin, teicoplanin and oxacillin. To assess this, we incubated VRSA strains with a subinhibitory concentration of the peptides for one hour. Afterward, the broth microdilution assay was used to determine the sensitivity of VRSA strains to antibiotics. Both peptides demonstrated the ability to re-sensitize VRSA strains to the effect of vancomycin, teicoplanin, and oxacillin. Pretreatment with subinhibitory concentrations of WR12 resulted in an 8- to 256-fold reduction in the MICs of vancomycin, teicoplanin, and oxacillin in the four VRSA strains tested (Table 4). Pretreatment of VRSA strains with sub-inhibitory concentrations of D-IK8 resulted in a 2- to 256-fold reduction in the MICs of vancomycin, teicoplanin, and oxacillin (Table 4). This study confirmed that, in addition to being used as antimicrobial agents alone or in combination with antibiotics in the treatment of staphylococcal infections, the peptides have the potential to suppress resistance of VRSA to conventional antibiotics. Clearly, further studies are needed to understand the mechanism of re-sensitization and their potential clinical applications. To explore the mechanism of re-sensitization, we monitored the leakage of preloaded calcein dye after exposure of VRSA to ½ × MIC of peptides for one hour. As demonstrated in Supplementary Figure S2, WR12 and D-IK-8 demonstrated leakage of calcein dye without affecting the survival of the VRSA strain (Supplementary Figure 2). This demonstrates that at subinhibitory concentrations, the peptides permeabilized the membrane potentially leading to increased access of antibiotics to their target. To confirm this hypothesis, VRSA (VRS10) was incubated with a sub-inhibitory concentration (½ × MIC) of WR12 and D-IK8 for one hour. Then treated with fluorescently labeled vancomycin (bodipy vancomycin) for 30 minutes. Bacterial pellets were fixed with 4% paraformaldehyde, and visualized under confocal microscope. As demonstrated in Supplementary Figure S3, pre-treatment of VRSA with WR12 and D-IK8 led to increased uptake and binding of fluorescently labeled vancomycin in contrast to non-peptide pre-treated samples. The fluorescence was maximized at the cell division septa and the cell wall of VRSA. Several studies reported that membrane acting antimicrobials re-sensitize resistant bacteria to antibiotics by similar mechanisms. For example, the protein-lipid complex from human milk, HAMLET (human alpha-lactalbumin made lethal to tumor cells) was able to resensitize MRSA to methicillin and VISA to vancomycin by depolarization of the bacterial membrane and dissipation of the proton motive force leading to more access and increased cell-associated binding of antibiotics^36^,^37^. Moreover, Minahk *et al*. reported that sub-lethal concentrations of enterocin synergize with antibiotics by dissipation of the proton motive force leading to inhibition of bacterial efflux systems and more accumulation of antibiotics intracellularly^38^. The amphibian antimicrobial peptide, esculentin-1b (1–18) was also reported to cause permeabilization of bacterial membranes at subinhibitory concentrations leading to synergism with conventional antibiotics against *E. coli*^39^. The peptidomimetic OAK (oligo-acyl-lysyl), was able to resensitize erythromycin-resistant *Escherichia coli* to erythromycin both *in vitro* and in an animal model of infection. The authors explained the mechanism of resensitization to be transient depolarization of the membrane potential at subinhibitory concentrations^40^.

### Toxicity of peptides on human keratinocytes

**Figure 3:**
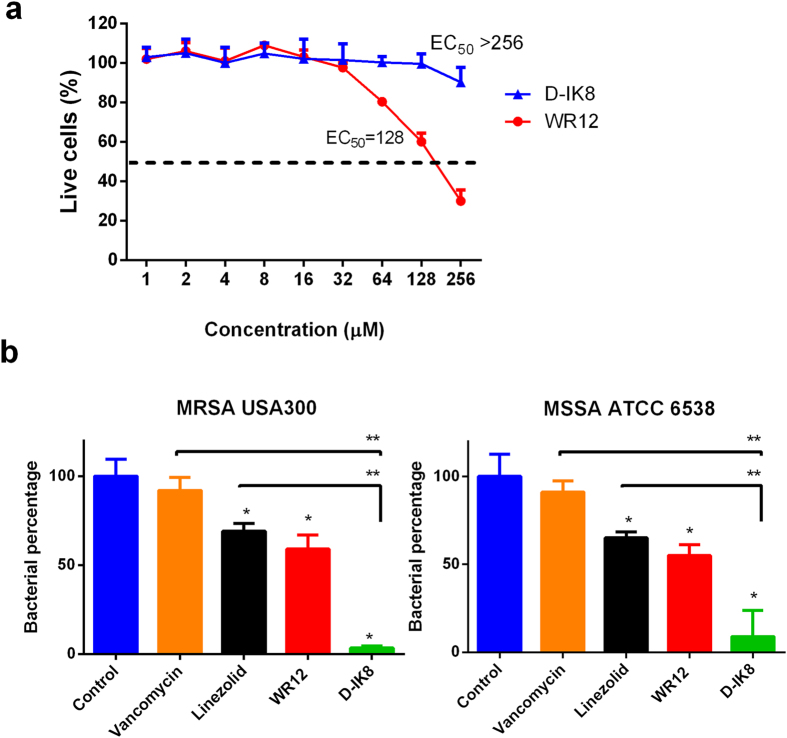
Toxicity (**a**) and intracellular anti-staphylococcal activity (**b**) of peptides in human keratinocyte (HaCat). (**a**) Cytotoxicity assay showing the percent mean absorbance at 490 nm after incubating human keratinocyte (HaCat) with peptides (WR12 and D-IK8) at different concentrations. Sterile water (peptide diluent) served as negative control. Cell viability was measured by MTS assay. EC_50_ is the half maximal effective concentration which equal 128 μM for WR12 and >256 μM for D-IK8. Results are expressed as means from three measurements ± standard deviation. **(b)** The effect of (WR12 and D-IK8) and antibiotics (vancomycin & linezolid) to kill MRSA USA300 (left panel) and MSSA ATCC 6538 (right panel) inside HaCat cells after treatment with 4 X MIC for 24 hr in DMEM + 10% FBS. Statistical analysis was calculated using one-way ANOVA, with post hoc Tukey’s multiple comparisons test. *P* values of <0.05 were considered significant. One asterisk (*) indicates significance from control negative. Two asterisk (**) indicates significance from control antibiotics. Detailed *P* values are listed: (**MRSA USA300**): vancomycin vs control, 0.3139; linezolid vs control, 0.0071; WR12 vs control, 0.0047; D-IK8 vs control, 0.00006; WR12 vs vancomycin, 0.0063; WR12 vs linezolid, 0.1336; D-IK8 vs vancomycin, 0.00003; D-IK8 vs linezolid, 0.00002; D-IK8 vs WR12, 0.00029. (**MSSA ATCC 6538**): vancomycin vs control, 0.3294; linezolid vs control, 0.0094; WR12 vs control, 0.0051; D-IK8 vs control, 0.0013; WR12 vs vancomycin, 0.0022; WR12 vs linezolid, 0.0712; D-IK8 vs vancomycin, 0.0009; D-IK8 vs linezolid, 0.0032; D-IK8 vs WR12, 0.0079. Experiments were done in three biological replicates per each treatment.

One of the major limitations for advancement of AMPs for clinical applications is toxicity to host tissues^1^. Here, we evaluated the cytotoxic effect of IDK-8 and WK12 on human keratinocytes (HaCat cells) using the MTS assay. The half maximal effective concentrations (EC_50_) of WR12 and D-IK8 were 128 and >256 μM, respectively (Fig. 3a). These values correlate to 64- and >32-fold of the MIC_50_ for WR12 and D-IK8, respectively. These results suggest that these AMPs have a favorable drug safety profile. The low toxicity of peptides against mammalian cells compared to their potent antimicrobial activity suggests their selective actions against the negatively charged bacterial membranes compared to the zwitterionic mammalian membranes.

### Intracellular antibacterial efficacy of peptides in human keratinocytes

Although *S. aureus* is not considered a typical intracellular pathogen, it can invade and thrive inside mammalian host cells. Moreover, treatment with conventional antimicrobials during the *S. aureus* intracellular invasion phase is a daunting task because most antimicrobials are unable to access intracellular replicative niches and achieve the optimum therapeutic concentrations within the infected cells^41^. Accordingly, treatment with conventional drugs of choice (such as vancomycin and aminoglycosides) is often associated with high clinical failures that exceed 40% in intracellular MRSA infections due to poor intracellular penetration of these drugs^42^,^43^. Since intracellular persistence of *S. aureus* constitutes a potent virulence component for various skin diseases such as impetigo and folliculitis^44^, we chose to assess the ability of AMPs to kill invasive intracellular MRSA infections. Due to the fact that MRSA and MSSA infect, reside, and replicate inside human keratinocytes, it was important to test the activity of WR12 and D-IK8 against MRSA and MSSA infected human keratinocytes. As depicted in Fig. 3b, both peptides showed significant reduction of intracellular MRSA and MSSA at 4 × MIC. D-IK8 displayed the most potent activity with significant reduction of 96% ± 1.15 and 91.08% ± 14.94 of intracellular MRSA and MSSA, respectively at 4 × MIC. WR12 at 4 × MIC demonstrated significant reductions of 40.98% ± 8.03 and 45% ± 6.23 of intracellular MRSA and MSSA, respectively. In contrast, antibiotics showed either low reduction (linezolid, 30–35%) or nonsignificant reduction (vancomycin) against intracellular staphylococci at 4 × MIC (Fig. 3b). Taken together, these results show that AMPs exhibits potent intracellular anti-staphylococcal efficacy in infected keratinocytes. These peptides, with their potent intracellular activity, could be useful in treating certain chronic exacerbating skin diseases such as Darier’s disease (keratosis follicularis) where *S. aureus* persists inside keratinocytes, leading to recurrent infections and treatment failure^44^. Interestingly, D-IK8 was more efficient than WR12 against MRSA and MSSA in infected keratinocytes compared to results obtained in pure culture. We hypothesized that the weak intracellular anti-staphylococcal activity of WR12 may be due to lack of intracellular penetration or due to reduced stability inside mammalian cells. To differentiate between the two possibilities, we conducted two experiments, (confocal study and stability study). Confocal studies of WR12-FITC demonstrated that this peptide penetrates and accumulates inside mammalian cells (Supplementary Figure S4). However, when WR12 was treated with trypsin for 4 hours, its anti-staphylococcal activity was abolished compared to D-IK8 (Supplementary Table S4). This supports our hypothesis that the weak intracellular anti-staphylococcal activity of WR12 may be due to the reduced stability of this peptide intracellularly.

### Efficacy of peptides in mice model of MRSA skin infection

**Figure 4:**
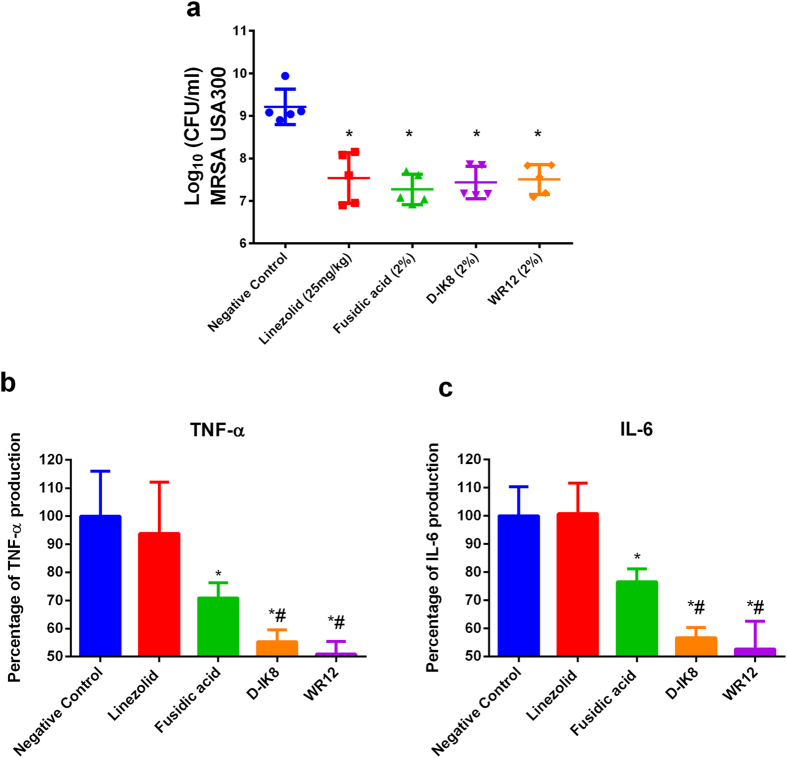
Efficacy of peptides and control antibiotics in bacterial load (**a**) and level of pro-inflammatory cytokines (**b,d**) in a murine model of MRSA skin infection. (**a**) Mouse was injected with the highly virulent MRSA USA300. After injection, mouse developed an abscess at the local site of injection over the back which further developed into an open wound within 48 hr from infection. Two days after the start of infection, mice were treated twice daily for 3 days either topically with fusidic acid (2%), D-IK8 (2%), WR12 (2%) formulated in petroleum jelly; or orally with linezolid (25 mg/kg). Petroleum jelly alone served as negative control. Figure 4a show the average log count of MRSA after treatment. Figure 4b,c show the effect of peptides on production of anti-inflammatory cytokines (TNF-α and IL-6) in MRSA skin lesions. Tissue homogenate supernatants were examined for cytokine production using ELISA. Cytokine levels were expressed as percent change relative to negative control. The two tailed Student *t* test, was used to determine statistical significance between two groups (a *P* value of < 0.05 was considered significant). One asterisk (*) indicates significance from control negative. Symbol (#) indicates statistically different than the antibiotic treated mice (*p* < 0.05). Detailed *P* values are listed: (**a**) linezolid vs control, 0.0009; fusidic acid vs control, 0.00005; D-IK8 vs control, 0.0001; WR12 vs control, 0.0001. (**b**) linezolid vs control, 0.6824; fusidic acid vs control, 0.0410; WR12 vs control, 0.0070; WR12 vs linezolid, 0.0168; WR12 vs fusidic acid, 0.0079; D-IK8 vs control, 0.0095; D-IK8 vs linezolid, 0.0235; D-IK8 vs fusidic acid, 0.0171. (**c**) linezolid vs control, 0.9328; fusidic acid vs control, 0.0230; WR12 vs control, 0.0046. WR12 vs linezolid, 0.0047; WR12 vs fusidic acid, 0.0188; D-IK8 vs control, 0.0024; D-IK8 vs linezolid, 0.0026; D-IK8 vs fusidic acid, 0.0040.

In light of our successful *in vitro* experiments, we moved forward with an *in vivo* experiment with a murine model of MRSA skin infection^45^,^46^,^47^,^48^,^49^. Briefly, groups of mice (n = 5) were injected with a highly virulent community-acquired MRSA strain USA300-0114. This clinical strain was isolated from a skin and soft-tissue outbreak in a state prison in Mississippi, USA. Twenty-four hours after the intradermal injection, mice developed an abscess at the injection site. Within 48 hours of infection, the abscess further developed into an open wound. Open wounds were treated topically with either 2% peptides, 2% fusidic acid, or vehicle alone (petroleum jelly). An additional group was treated orally with 25 mg/kg linezolid. All groups of mice were treated twice daily for three days. As presented in Fig. 4a, all treatments significantly reduced the mean bacterial counts of MRSA in wounds compared to the control group (*P* ≤ 0.05). WR12 and D-IK8 showed 1.71 ± 0.07 and 1.78 ± 0.07 log reduction of MRSA USA300, respectively. The group treated with fusidic acid produced a 1.94 ± 0.352 log reduction in bacterial count. The group treated with oral linezolid generated a 1.678 ± 0.38 log reduction in bacterial load. These results reveal that our peptides are very effective in reducing the bacterial load in MRSA skin lesions.

The clinical severity of skin infections caused by *S. aureus* is driven by excess production of host pro-inflammatory cytokines more so than by bacterial burden. In addition, excessive host inflammation delays the wound healing process and leads to more scar formation^50^,^51^. We hypothesized that therapeutics with combined antibacterial and anti-inflammatory properties should be superior to traditional antibiotics for treatment of *S. aureus* skin infections. To confirm, we investigated the immune-modulatory effect of our peptides by measuring the levels of pro-inflammatory cytokines produced normally during infection, including tumor necrosis factor-α (TNF-α) and interleukin-6 (IL-6)^52^. As shown in Fig. 4b,c, both peptides produced a significant reduction in TNF-α and IL-6 levels compared to the untreated control and antibiotic-treated groups. WR12 and D-IK8 reduced the TNF-α level by 50% and 44%, respectively, while the IL-6 level was reduced by 48% and 42%, respectively. However, treatment with 2% fusidic acid reduced TNF-α and IL-6 levels by only 29% and 25%, respectively, which is in agreement with previous findings^53^. Linezolid, on the other hand, did not show any significant reduction in the levels of cytokines when compared to the control group. The combined antimicrobial and immunomodulatory effects of the AMPs should confer an added advantage in the treatment of *S. aureus* skin infection and might help in promoting epithelization and accelerating wound healing processes. Some AMPs have reached preclinical and clinical phases for topical treatment of bacterial infection. One of the AMPs that has advanced furthest in clinical trials is pexiganan for curing diabetic foot ulcers^54^. Topical application of 2% pexiganan cream showed therapeutic resolution equivalent to oral ofloxacin for treatment of mild infections of diabetic foot ulcers^54^. Interestingly, no significant resistance to pexiganan emerged among patients who received pexiganan. However, bacterial resistance to ofloxacin emerged in some patients who received ofloxacin^54^. Currently, pexiganan is undergoing phase 3 development as a topical agent for treatment of mild infections of diabetic foot ulcers (ClinicalTrials.gov registration numbers NCT01594762 and NCT01590758). Another compound that showed success in clinical trials is the peptidomimetic, brilacidin. Brilacidin is a defensin-mimetic that targets the bacterial membrane, similar to AMPs. The MIC_90_ of brilacidin against a collection of multidrug-resistant *S. aureus* isolates was 2 μg/ml. Brilacidin demonstrated clinical efficacy and safety on two studies of phase II clinical trials for the treatment of acute bacterial skin and skin structure infections (ABSSSI). The FDA approved brilacidin to advance into Phase III clinical trials^55^. Lytixar (LTX-109) is a synthetic, membrane-degrading peptide that has been developed by Lytix Biopharma (Oslo); this peptide has completed a Phase I/IIa clinical trial for nasally colonized MRSA. A significant effect on nasal decolonization of MRSA and MSSA was observed after only two days of LTX-109 treatment in subjects treated with 2% or 5% LTX-109, compared to the vehicle. The success of pexiganin, brilacidin, and LTX-109 demonstrates the promise that antimicrobial peptides have as potential therapeutic agents for treatment of multidrug-resistant pathogens.

## Conclusion

We have successfully demonstrated the potential utility of WR12 and D-IK8 against MRSA and VRSA clinical isolates. WR12 and D-IK8 were superior to antibiotics, demonstrating potent and rapid eradication of persister cells and MRSA cells in stationary phase of growth. Moreover, WR12 and D-IK8 disrupted the mature biofilms of staphylococci and were able to kill intracellular staphylococci at a more significant rate than antibiotics of choice. Additionally, WR12 and D-IK8 augmented the antibacterial action of topical and systemic antibiotics, which will increase the clinical uses of these antibiotics and decrease their adverse effects. Finally, WR12 and D-IK8 significantly reduced the count of MRSA in skin lesions and displayed potent immunomodulatory effects. Collectively, the qualities of WR12 and D-IK8 presented here have the potential to be used for different clinical applications, including resilient MRSA infections.

## Materials and Methods

### Antibacterial assays

The minimum inhibitory concentrations (MIC) of peptides and antibiotics were determined by the broth microdilution technique according to the guidelines of the Clinical and Laboratory Standards Institute (CLSI)^56^. MIC assays were carried out with an initial bacterial inoculum of 5 × 10^5^ colony forming units (CFU/ml) in MHB. Peptides and antibiotics were added to polystyrene 96-well plates at desired concentrations. MHB were supplemented with 50 mg/liter Ca^2^ in case of daptomycin. MIC was defined as the lowest concentration of peptide or antibiotic which inhibited the visible growth of bacteria.

### Time kill assay

MRSA USA300 was grown overnight in MHB, then diluted in fresh MHB and incubated aerobically at 37 °C until bacteria reached logarithmic phase of growth (OD_600_ = 0.2). Then bacteria were diluted to 5.6 × 10^6^ CFU/ml in MHB. Peptides (at 5 × and 10 × MIC) and antibiotics at 10 × MIC were added to diluted bacteria, and incubated aerobically at 37 °C in a shaking incubator at 250 r.p.m. Aliquots at specified time points were taken, serially diluted in phosphate-buffered saline (PBS) and plated, in triplicate, on TSA. CFUs were counted after incubation of plates for 24 hours at 37 °C. The kinetics of killing against stationary phase bacteria was done as described previously^22^,^25^,^26^. MRSA USA300 was grown in MHB with aeration at 250 r.p.m. at 37 °C overnight. Then bacteria were exposed to peptides and antibiotics at 10 × MIC. Aliquots were taken and plated as described above. Formation of persister cells was done as described before^22^,^25^,^26^. Briefly, MRSA USA300 was grown overnight in MHB, then diluted in fresh MHB and incubated until cells reached logarithmic phase of growth. Bacteria were then exposed to10 × MIC ciprofloxacin for 6 hours. Peptides and antibiotics, at 10 × MIC, were added to bacteria after 6 hours from ciprofloxacin treatment. Bacteria were incubated aerobically at 37 °C in a shaking incubator at 250 r.p.m. Aliquots at specified time points were taken and counted as described above.

### Efficacy of peptides on *Staphylococcus* biofilms

The efficacy of peptides to disrupt biofilms was conducted as described before^57^. Briefly, isolates of *S. aureus* (ATCC 6538) and *S. epidermidis* (ATCC 35984) grown overnight were diluted 1:100 in TSB + 1% glucose and incubated in 96-well plates at 37 °C for 24 hours or 48 hours. After removing media, wells were rinsed with PBS to remove planktonic bacteria before re-filling wells with fresh MHB. Peptides and antibiotics were added at desired concentrations and plates were incubated at 37 °C for 24 hours. After incubation, wells were washed and biofilms were stained with 0.5% (w/v) crystal violet for 30 minutes. The dye was solubilized with ethanol (95%) and the optical density (OD) of biofilms was measured.

### Combination therapy analysis

The synergistic effect between peptides and antibiotics was assessed by the combination assay as described previously^57^,^58^. Two-fold serial dilutions of antimicrobials (WR12, D-IK8, and antibiotics) were tested in the presence of a fixed concentration of peptide equal to ¼ × peptide MIC, which did not inhibit the growth of bacteria alone. The fractional inhibitory concentration (FIC) index was calculated as follows: FIC of drug A = MIC of drug A in combination/MIC of drug A alone, FIC of drug B = MIC of drug B in combination/MIC of drug B alone, and FIC index = FIC of drug A + FIC of drug B. An FIC index of ≤0.5 was classified as synergism. Additive was defined as an FIC index of 1. Antagonism was defined as an FIC index of >4.

### Re-sensitization of VRSA to vancomycin, teicoplanin and oxacillin in the presence of subinhibitory concentration of peptides

Resensitization of VRSA strains to antibiotics (vancomycin, teicoplanin and oxacillin) was done as described previously^59^. Briefly, ½ × MIC of WR12 and D-IK8 was incubated with VRSA (bacterial inoculum of 5 × 10^5^ colony forming unit (CFU/ml)) in MHB at room temperature for 60 minutes. After incubation, peptide-treated bacteria were added in 96-well plates. Antibiotics, at a concentration equal to their MIC, were added to the first row and diluted. Bacteria treated with ½ × MIC of WR12 and D-IK8 served as the negative control as we didn’t observe bacterial inhibition at this concentration. The plate was incubated for 24 hours at 37 °C and the MIC was recorded.

### Toxicity of peptides on human keratinocytes

Peptides were assayed for potential *in vitro* toxicity against human keratinocytes (HaCaT) as described before^60^. Briefly, cells were seeded at a density of 1.5 × 10^4^ per well in a tissue culture 96-well plate (CytoOne, CC7682-7596) in DMEM supplemented with 10% fetal bovine serum (FBS), and incubated at 37 °C in a 5% CO_2_ atmosphere for 24 hours. The cells were treated with compounds at different concentrations for 24 hours. After incubation, the cells were washed and incubated with 100 μL of DMEM media containing 20 μL of MTS reagent for 4 hours at 37 °C. Corrected absorbance readings were taken using an ELISA microplate reader (Molecular Devices, Sunnyvale, CA, USA).

### Intracellular antibacterial efficacy of peptides in human keratinocytes

Infection of human keratinocytes (HaCaT) was done as described previously^44^. Briefly, HaCaT cells were seeded and incubated as described above. Following incubation, the cells were infected either with methicillin-sensitive *S. aureus* ATCC 6538 or methicillin resistant *S. aureus* USA300, (at a multiplicity of infection 50:1) in DMEM + 10% FBS for 2.5 hours. After infection, the wells were washed with 200 μl media with lysostaphin (10 μg/ml) and further incubated for 30 minutes with lysostaphin to kill any remaining extracellular bacteria. Drugs were diluted in DMEM + 10% FBS to the desired concentrations (4 × MIC) and wells were treated with 100 μl of DMEM + 10% FBS containing drugs for 24 hours. Medium alone was used as a negative control. After incubation, the media were aspirated and washed twice with PBS to remove any residual drugs. HaCaT cells were lifted with trypsin. Then 100 μl of PBS with 0.01% triton X was added in each well to lyse HaCaT cells. Subsequently, bacteria were diluted and plated on TSA plates. Plates were incubated at 37 °C for 24 hours. After incubation, bacteria were counted and analyzed. Experiments were repeated twice independently and the average was reported.

### Efficacy of peptides in mice model of MRSA skin infection

The animal care and all experiments were approved and performed in accordance with the guidelines approved by Purdue University Animal Care and Use Committee (PACUC). Female BALB/c mice (6–8 weeks old) were obtained from Harlan Laboratories, Indianapolis, IN. All procedures were approved by the Purdue University Animal Care and Use Committee (PACUC) (protocol no: 1207000676). The murine model of MRSA skin infection was done as described previously^45^,^46^,^47^,^48^,^49^. Briefly, the posterior upper backs of mice were shaved and mice were injected intradermal by 40 μl of MRSA USA300 (3 × 10^7^ CFU/40 μl) in sterile phosphate-buffered saline (PBS) using a 27-gauge insulin syringe. Mice were randomly divided into five groups and each group contained five animals. Forty-eight hours after infection and formation of open wound, groups of mice were treated topically either with 2% fusidic acid, 2% WR12 or 2% D-IK8 formulated in 20 mg petroleum jelly. One group received vehicle only (petroleum jelly) and the last group was treated orally with linezolid (25 mg/kg). All groups were treated twice a day for 3 days. Twenty-four hours after the last treatment, mice were euthanized, and the skin lesion was removed and homogenized in 1 ml tryptic soy broth. Samples were diluted, plated in mannitol salt agar in triplicate, and incubated aerobically at 37 °C. After 24 hours incubation, the colony forming units (CFU) were counted. Cytokine detection of tumor necrosis factor-α (TNF-α) and interleukin-6 (IL-6) in skin lesions was done using ELISA as described before and according to manufacturer instructions^45^. Cytokine levels were expressed as percent change relative to negative control.

### Statistical analyses

Statistical analyses were performed using GraphPad Prism 6.0 software (GraphPad Software, La Jolla, CA, USA). Comparison between two groups were analyzed using two-tailed unpaired Student t-tests. Comparison between three groups or more were analyzed using one-way ANOVA, with post hoc Tukey’s multiple comparisons test. *P*-values of <0.05 were considered significant.

## Additional Information

**How to cite this article**: Mohamed, M. F. *et al*. Evaluation of short synthetic antimicrobial peptides for treatment of drug-resistant and intracellular *Staphylococcus aureus*. *Sci. Rep.* **6**, 29707; doi: 10.1038/srep29707 (2016).

## Supplementary Material

Supplementary Information

## Figures and Tables

**Table 1:** Minimum inhibitory concentration (MIC) of peptides and antibiotics against clinical and drug-resistant staphylococci isolates.

Strain Type^a^	Strain ID	MIC (μM)								
		WR12	D-IK8	LL-37	Linezolid	Vancomycin	Erythromycin	Kanamycin	Ciprofloxacin	Trimethoprim
**MSSA**	**ATCC 6538**	4	8	16	2	0.5	≤0.25	4	≤0.25	1
	**RN4220 (NRS107)**	2	8	>128	1	0.5	≤0.25	2	≤0.25	1
	**NRS77**	2	8	>128	2	0.5	≤0.25	1	≤0.25	1
	**NRS846**	16	16	>128	4	0.5	>128	4	≤0.25	2
	**NRS860**	4	16	>128	1	0.5	>128	4	16	2
**MRSA**	**USA100 (NRS382)**	2	8	>128	4	0.5	>128	32	8	1
	**USA200 (NRS383)**	4	8	>128	4	0.5	32	>128	>128	>128
	**USA300-0114 (NRS384)**	4	16	>128	2	0.5	>128	>128	4	2
	**USA400 (NRS123)**	2	8	>128	4	0.5	≤0.25	1	≤0.25	4
	**USA500 (NRS385)**	8	16	>128	4	1	>128	>128	32	>128
	**USA700 (NRS386)**	2	8	>128	4	1	8	>128	0.5	2
	**USA800 (NRS387)**	2	16	>128	4	0.5	≤0.25	1	≤0.25	2
	**USA1000 (NRS483)**	2	8	>128	4	0.5	2	4	2	4
	**USA1100 (NRS484)**	1	8	>128	4	1	≤0.25	1	0.5	2
	**NRS194**	2	8	>128	4	1	≤0.25	1	2	≤0.25
	**NRS108**	1	8	>128	4	0.5	32	>128	4	>128
	**NRS119 (Lin**^**R**^)	4	16	>128	128	0.5	≤0.25	>128	>128	>128
	**ATCC 43300**	2	4	>128	4	0.5	>128	>128	≤0.25	2
	**ATCC** **BAA-44**	4	8	>128	4	0.5	>128	>128	8	1
	**NRS70**	1	4	>128	2	0.5	>128	32	≤0.25	2
	**NRS71**	4	8	>128	2	0.5	>128	>128	>128	4
	**NRS100**	8	16	>128	2	0.5	>128	4	≤0.25	2
	**NRS123**	2	8	>128	2	0.5	≤0.25	1	≤0.25	4
**VISA**	**NRS1**	1	8	>128	2	8	>128	>128	16	2
	**NRS19**	1	8	>128	2	4	>128	>128	>128	>128
	**NRS37**	1	8	>128	2	8	>128	>128	32	2
**MRSE**	**NRS101**	4	4	16	2	0.5	>128	>128	≤0.25	>128

^a^(MSSA): methicillin sensitive *Staphylococcus aureus;* (MRSA): methicillin resistant *Staphylococcus aureus*; (VISA): vancomycin intermediate *Staphylococcus aureus* and (MRSE): methicillin resistant *Staphylococcus epidermidis*.

**Table 2:** Minimum inhibitory concentration (MIC) of peptides and antibiotics against clinical vancomycin resistant *Staphylococcus aureus* (VRSA) isolates.

Strain ID	MIC(μM)						
	WR12	D-IK8	LL-37	Vancomycin	Oxacillin	Kanamycin	Teicoplanin
**VRS4**	4	8	>128	32	64	>128	16
**VRS5**	8	16	>128	32	4	>128	8
**VRS10**	8	16	>128	>128	>128	>128	>128
**VRS11a**	4	16	>128	>128	>128	>128	>128
**VRS11b**	4	16	>128	>128	>128	>128	>128
**VRS12**	8	16	>128	>128	>128	>128	>128
**VRS13**	4	16	>128	>128	>128	>128	>128

**Table 3:** The fractional inhibitory concentration index range of peptides in combination with each other and with antibiotics against *Staphylococcus aureus* isolates.

Strain	∑FICI^a^											
		WR12	D-IK8	Fusidic acid	Mupirocin	Daptomycin	Teicoplanin	Vancomycin	Linezolid	Ciprofloxacin	Meropenem	Oxacillin
**MSSA (ATCC 6538)**	**WR12**	–	0.27	0.31	0.28	0.31	nd	0.26	0.28	0.50	0.28	0.27
	**D-IK8**	0.31	–	0.31	0.50	0.31	0.31	0.75	1.25	1.25	0.50	0.38
**MRSA (USA300)**	**WR12**	–	0.31	0.38	0.27	0.31	0.28	0.27	0.27	0.31	0.26	0.31
	**D-IK8**	0.38	–	0.50	0.75	0.31	0.75	0.75	0.75	1.25	1.25	1.25
**MRSE (NRS101)**	**WR-12**	–	0.38	0.38	0.38	0.26	0.31	0.38	0.50	0.75	0.75	0.28
	**D-IK8**	0.31	–	0.38	0.75	0.26	0.50	1.25	0.50	0.75	1.25	0.75
**VRSA (VRS10)**	**WR12**	–	1.25	0.50	0.75	0.26	0.50	0.27	1.25	0.75	0.38	0.75
	**D-IK8**	0.75	–	0.50	0.75	0.26	0.75	0.27	1.25	1.25	1.25	1.25

^a^**∑**FICI, fractional inhibitory concentration index. FIC index was interpreted as follows: An FIC index of ≤0.5 is considered to demonstrate synergy. Additive was defined as an FIC index of 1. Antagonism was defined as an FIC index of >4. nd, not determined. (MSSA): methicillin sensitive *Staphylococcus aureus;* (MRSA): methicillin resistant *Staphylococcus aureus*; (VRSA): vancomycin resistant *Staphylococcus aureus* and (MRSE) methicillin resistant *Staphylococcus epidermidis.*

**Table 4:** Resensitization of vancomycin resistant *S. aureus* (VRSA) to vancomycin, teicoplanin and oxacillin using a sub-inhibitory concentration (½ × MIC) of WR12 or D-IK8.

Strain	Fold of re-sensitization^a^			
		Vancomycin	Teicoplanin	Oxacillin
**VRS4**	**WR12**	8	32	128
	**D-IK8**	2	32	No sensitization effect.
**VRS10**	**WR12**	256	256	256
	**D-IK8**	256	256	256
**VRS12**	**WR12**	128	8	256
	**D-IK8**	64	No sensitization effect.	256
**VRS13**	**WR12**	64	16	256
	**D-IK8**	128	32	256

^a^Fold of re-sensitization: it is the ratio of the MIC of antibiotic alone divided by the MIC of antibiotic after re-sensitization with (½ × MIC) of peptides (WR12 or D-IK8).
