# Supplementary material for: Evaluation of short synthetic antimicrobial peptides for treatment of drug-resistant and intracellular Staphylococcus aureus
Source: Sci Rep. 2016 Jul 11;6:29707. doi: 10.1038/srep29707 (PMC4942614; doi:10.1038/srep29707)
Supplement: Supplementary Information [file srep29707-s1.pdf]

Supplementary materials

Evaluation of short synthetic antimicrobial peptides for treatment of drug-resistant and intracellular *Staphylococcus aureus*

Mohamed F. Mohamed[1], Ahmed Abdelkhalek[1], and Mohamed N. Seleem[1,2]*

[1] Department of Comparative Pathobiology, College of Veterinary Medicine, Purdue University, West Lafayette, IN 47907, USA

[2] Purdue Institute for Inflammation, Immunology, and Infectious Disease, Purdue University, West Lafayette, IN 47907, USA

Supplementary Materials and methods:

Bacterial isolates, peptides and reagents

Methods were carried out in accordance with approved guidelines. Clinical isolates of *Staphylococci* are presented in (supplementary table S1). Peptides, WR12 (RWWRWWRRWWRR), D isoform of IK8 (irikirik) ("here we refer to it as "D-IK8"), LL-37 (LLGDFFRKSKEKIGKEFKRIVQRIKDFLRNLVPRTES) and pexiganan (GIGKFLKKAKKFGKAFVKILKK) were synthesized by GenScript (Piscataway, NJ) using solid-phase 9-fluorenylmethoxy carbonyl (Fmoc) chemistry and purified to a purity of 95% using reverse-phase high-performance liquid chromatography (HPLC). Peptide mass was confirmed by mass spectrometry (supplementary materials). Antibiotics were purchased from commercial vendors, vancomycin hydrochloride (Gold Biotechnology, St. Louis, MO), linezolid (Selleck Chemicals, Houston, TX), clindamycin (TCI chemicals, Portland, OR), erythromycin, gentamicin were purchased from Sigma-Aldrich (St. Louis, MO). Fetal bovine serum (FBS) was purchased from Sigma-Aldrich (St. Louis, MO). Dulbecco's Modified Eagle's medium (DMEM) was

purchased from Life technologies. MTS reagent was purchased from Promega (Madison, WI). Mueller-Hinton broth (MHB) was purchased from Sigma-Aldrich, while Trypticase soy broth (TSB) and Trypticase soy agar (TSA) were purchased from Becton-Dickinson, Cockeysville, MD. Mannitol salt agar (MSA) was purchased from Hardy Diagnostics (Santa Maria, CA). Enzyme-linked immunosorbent assay (ELISA) development kits for cytokines detection were purchased from R&D Systems, Inc. (Minneapolis, MN).

Antibacterial assays in presence of salts:

To investigate the activity of peptides in the presence of high salt concentrations, WR12, D-IK-8 and pexiganan were tested against MRSA USA300 in a cation-adjusted MHB or in MHB with added concentrations of NaCl (150 mM) or $MgCl_2$ (2 mM). The MIC was subsequently identified, as described before and according to the guidelines of the Clinical and Laboratory Standards Institute (CLSI) [1]

To determine the effect of protease digestion on the antimicrobial activity of the peptides, each peptide was first incubated with trypsin at a molar ratio of 500:1 (peptide: enzyme) in the digestion buffer (50 mM Tris–HCl, pH 7.4) at 37°C for 4 hours. After incubation, the digestion mixture was heated at 80°C for 10 min to halt the enzyme reaction. After these treatments, the procedures conducted were the same as the MIC assay described above.

Membrane permeabilization assay (Calcein leakage assay)

Membrane permeabilization of *S. aureus* by peptides was monitored and quantified by the leakage of the preloaded fluorescent dye, calcein, as described before[2]. MRSA USA300 and VRS10 was

grown in MHB to logarithmic phase at 37°C. Cells were then harvested by centrifugation, washed twice with PBS, and then adjusted spectrophotometrically to an OD_{600} of 1.0 (≈10^9 CFU/ml) in PBS containing 10% (vol/vol) MHB. Then MRSA cells were incubated with 3 µM calcein AM for 1 hr at 37°C. Calcein-loaded cells were harvested by centrifugation (3,000 × *g*, 10 min), suspended in PBS, and diluted to achieve a final inoculum of 10^7 CFU/ml. Aliquots of 100 µL were then added into a sterile black-wall 96-well plate. In case of MRSA USA300, WR12 and D-IK8 were added in concentrations equivalent to 5 × and 10 × MIC. In case of VRS10, WR12 and D-IK8 were added in concentrations equivalent to 0.5 × MIC and incubated for 1 hr. Bacteria treated with peptide diluent (sterile water) served as negative controls. Calcein leakage was measured for 120 min using a fluorescence plate reader (FLx800 model BioTek® Instruments, Inc. Winooski, Vermont). Membrane permeabilization (%) was calculated as the absolute percent calcein leakage by peptides with respect to calcein-loaded with no-peptide treated cells. Experiments were done in triplicate and repeated independently twice.

Confocal Microscopy : Uptake of WR12-FITC in mammalian cells

J774A.1 cells were seeded at a density of 1.5 x 10^5 cells/well in a 4-well Lab-Tek chambered slides in DMEM media supplemented with 10% fetal bovine serum (FBS), and incubated at 37°C in a 5% CO_2 atmosphere for 20 hours. The media were aspirated and the cells were washed 1X with 400 µL PBS. Following incubation, the cells were washed once with DMEM media. Then the cells were incubated with WR12-FITC (10 µM) for 3 hours at 37 °C and 5% CO_2. The cells were washed 3X with PBS and visualized under 60X oil objective of Nikon A1R multi-photon inverted confocal microscope.

Sub-inhibitory concentration of WR12 and D-IK8 increase the uptake and binding of bodipy vancomycin

We investigated the binding and association of fluorescently labeled vancomycin (bodipy vancomycin) with VRSA a previously described[3,4]. Briefly, VRS10 was incubated with sub-inhibitory concentration of WR12 and D-IK8 (0.5 X MIC) for one hour and then treated with bodipy vancomycin (16 µg/ml) for 30 minutes. Samples treated with bodipy vancomycin only served as a control. After incubation, bacteria were centrifuged at 9,000×*g* for 5 min, washed four times with PBS, and resuspended in a small volume of PBS. Bacterial pellets were fixed with 4% paraformaldehyde, and visualized under 40X oil objective of Nikon A1R multi-photon inverted confocal microscope.

Supplementary table S1: Clinical isolates of Staphylococci strains used in the study

Strain type	Strain ID	Isolation	Molecular Typing		Phenotypic Properties
		Origin	SCC*mec* type	*spa* type	
Methicillin sensitive *Staphylococcus aureus* (MSSA)	ATCC 6538	-	-	-	Quality control and biofilm forming strain
	NRS107, RN4220	United States	-	YHGGFMBQBLO	Resistant to mupirocin
	NRS77 (RN1)	United Kingdom	-	YHGGFMBQBLO	produce alpha, delta and gamma-hemolysins, Genome

					sequenced strain
	NRS846 (VCU006)	-	-	-	Genome sequenced strain
	NRS860 (VCU089)	-	-	-	Genome sequenced strain
Methicillin resistant *Staphylococcus aureus* (MRSA)	NRS382, USA100	United States (Ohio)	II	TJMBMDMGMK	Resistant to ciprofloxacin, clindamycin, erythromycin
	NRS383, USA200	United States (North Carolina)	II	WGKAKAOMQQQ	Resistant to ciprofloxacin, clindamycin, erythromycin, gentamicin, and methicillin
	NRS384, USA300-0114	United States (Mississippi)	IV	YHGFMBQBLO	Resistant to erythromycin, methicillin, and tetracycline
	NRS123, USA400	United States (North Dakota)	IV	UJJFKBPE	Resistant to methicillin and tetracycline
	NRS385, USA500	United States (Connecticut)	IV	YHGCMBQBLO	Resistant to ciprofloxacin, clindamycin, erythromycin, gentamicin, methicillin, tetracycline, and trimethoprim
	NRS386, USA700	United States (Louisiana)	IV	UJGFMGGM	Resistant to erythromycin

					and methicillin
	NRS387, USA800	United States (Washington)	IV	TJMBMDMGGMK	Resistant to methicillin
	NRS483, USA1000	United States (Vermont)	IV	-	Resistant to erythromycin and methicillin
	NRS484, USA1100	United States (Alaska)	IV	-	Resistant to methicillin
	NRS194, C1999000529	United States (North Dakota)	IV	UJFKKPFKPE	Resistant to methicillin
	NRS108, A960649	France	I	YHGFMMBQBLO	Resistant to gentamicin
	NRS119, SA LinR #12	United States (Massachusetts)	IV	YHGCMBQBLO	Resistant to linezolid
	ATCC 43300	United States (Kansas)	-	-	Resistant to methicillin
	ATCC BAA-44	Lisbon, Portugal	I	YHFGFMBQBLO	Multidrug resistant strain.
	NRS70 (N315)	Japan	II	TJMBMDMGMK	Resistant to erythromycin and spectinomycin
	NRS71	United Kingdom			Resistant to tetracycline and methicillin
	NRS100 (COL)	United Kingdom	I	YHGFMBQBLO	Resistant to tetracycline and methicillin, genome sequenced

	NRS123	United States (North Dakota)			Resistant to tetracycline and methicillin
Vancomycin intermediate *Staphylococcus aureus* (VISA)	NRS1 ATCC 700699	Japan	II	TJMBMDMGMK	Resistant to aminoglycosides and tetracycline Glycopeptide-intermediate *Staphylococcus aureus*
	NRS19, HIP07256	United States (Illinois)	II	TJMBMDMGMK	Glycopeptide-intermediate *Staphylococcus aureus*
	NRS37, LIM 3	France	I	YHFGFMBQBLO	Glycopeptide-intermediate *Staphylococcus aureus*
Vancomycin Resistant *Staphylococcus aureus* (VRSA)	VRS4	United States	-	-	Resistant to vancomycin
	VRS5	United States	-	-	Resistant to vancomycin
	VRS10	United States	-	-	Resistant to vancomycin
	VRS11a	United States	-	-	Resistant to vancomycin
	VRS11b	United States	-	-	Resistant to vancomycin
	VRS12	United States	-	-	Resistant to vancomycin

	VRS13	United States	-	-	Resistant to vancomycin
Methicillin resistant *Staphylococcus epidermidis* (MRSE)	ATCC 35984 NRS101	United States	-	-	Prototype biofilm producer, Resistant to methicillin, kanamycin and gentamicin

Supplementary table S2: Amino acid sequence and physicochemical properties of peptides used in this study

Peptide designation	Amino acid sequence[a]	Length	Molecular weight	Charge	Hydrophobic amino acids
WR-12	RWWRWWRRWWRR	12	2072.4	+ 6	50 %
D-IK8	irikirik	8	1040.28	+ 4	50 %
Pexiganan	GIGKFLKKAKKFGKAFVKILKK	22	2478.163	+9	45%

[a]Small underlined residues represent D-amino acids

Supplementary table S3: Comparison of the minimum inhibitory concentration (MIC) of peptides compared to pexiganan against four *Staphylococcus* isolates:

	MIC (μM)				
Strain Type	Strain ID	WR12	D-IK8	LL-37	Pexiganan
MSSA	ATCC 6538	4	8	16	16
MRSA	USA300	4	8	>128	16
VRSA	VRS10	8	16	>128	32
MRSE	NRS101	4	4	16	1

Supplementary table S4: Minimum inhibitory concentration (MIC) of peptides against Methicillin resistant *Staphylococcus aureus* (MRSA) USA300 in different media conditions:

	MIC (μM)				
	MHB	Cation adjusted MHB	NaCl 150 mM	$MgCl_2$ 2 mM	Trypsin 1:500
WR12	4	4	4	8	> 64
D-IK8	16	32	64	64	16
Pexiganan	16	32	64	16	nd

nd: not determined

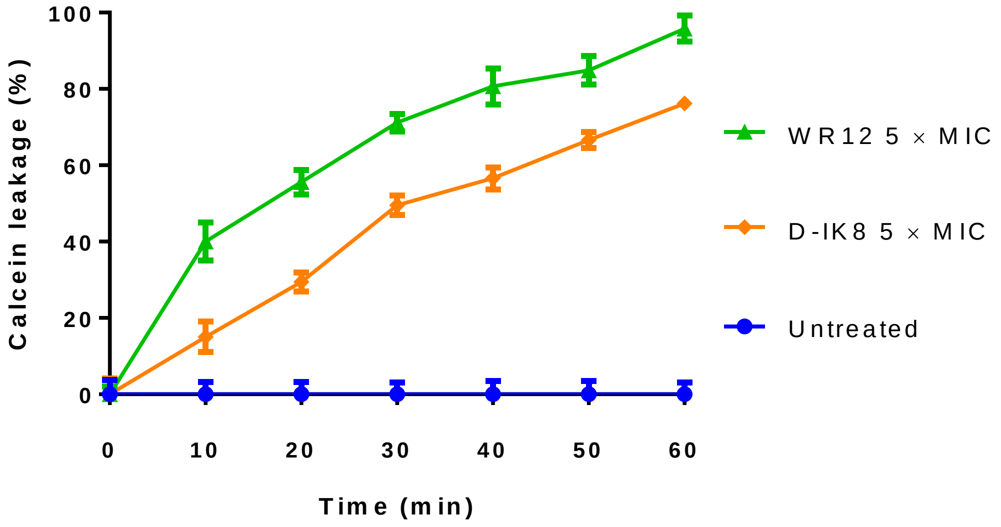

Supplementary figure 1: Permeabilization of the cytoplasmic membrane of MRSA USA300 as a function of peptide concentration, indicated by percent of calcein leakage for 60 min exposure. The results are given as means ± SD (n = 3; data without error bars indicate that the SD is too small to be seen).

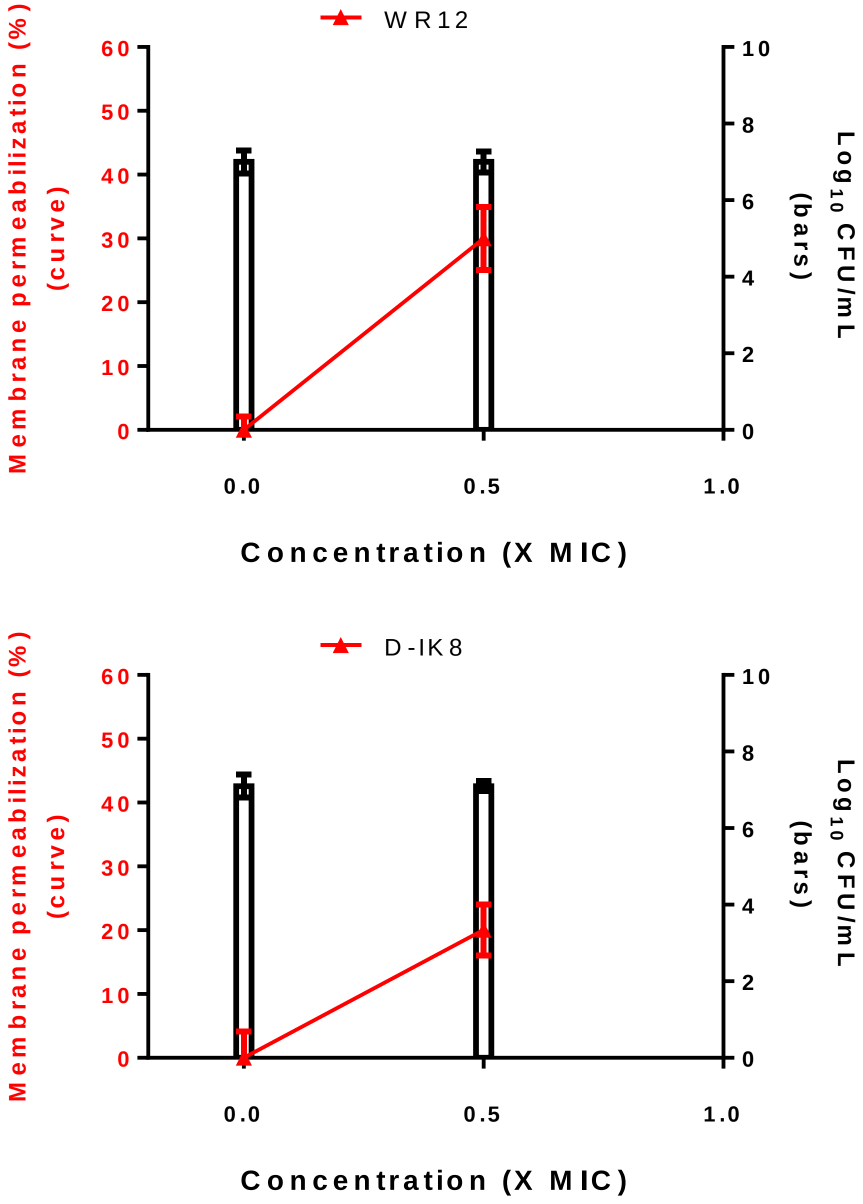

Supplementary figure 2: Survival and permeabilization of the cytoplasmic membrane of VRS10 after treatment with 0.5 X MIC of WR12 and D-IK8. Membrane permeabilization is indicated by percent of calcein leakage after 60 min exposure to peptides. Bacterial count of VRS10 is presented as columns. The results are given as means ± SD (n = 3; data without error bars indicate that the SD is too small to be seen).

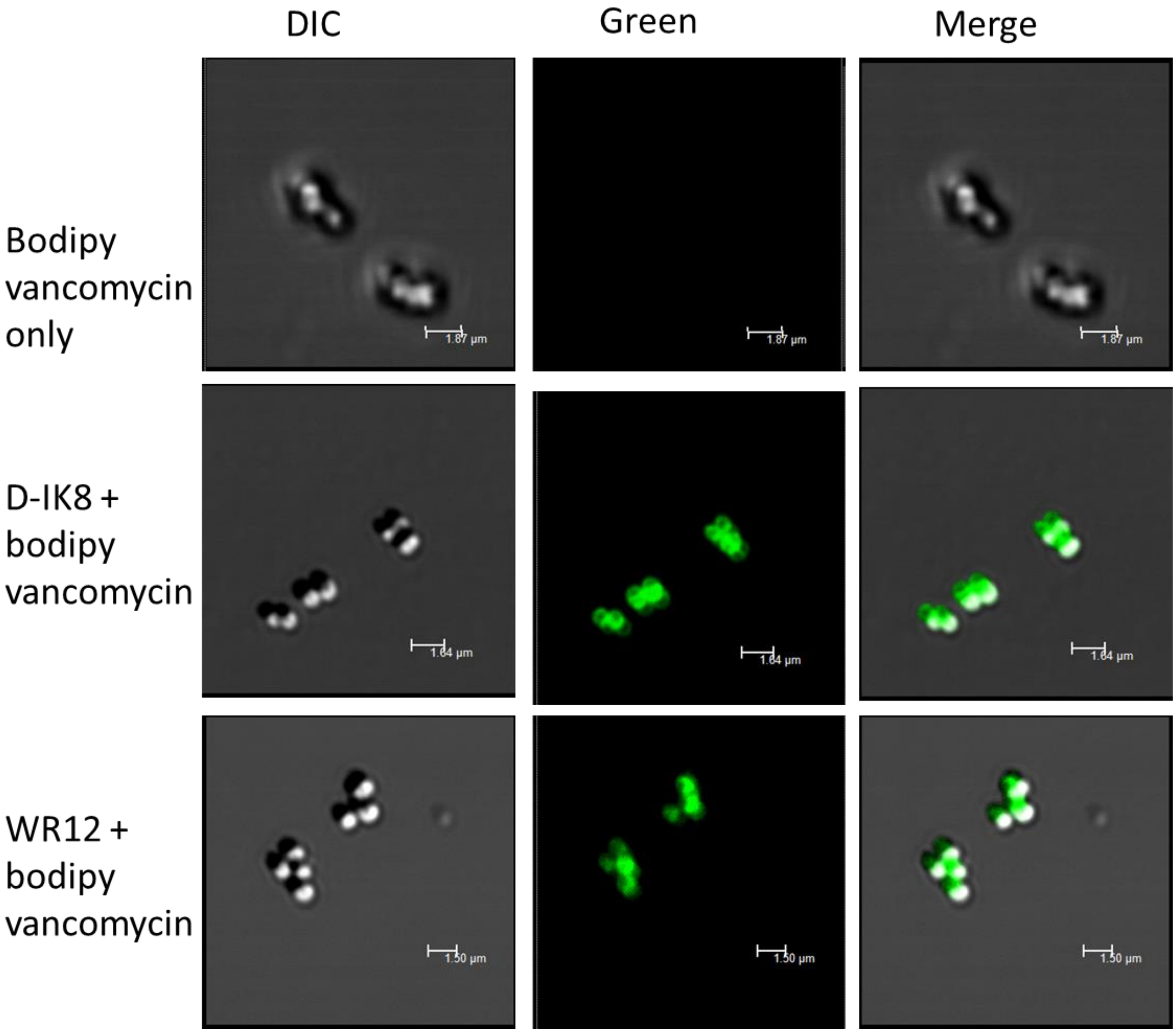

Supplementary figure 3: Sub-inhibitory concentration of WR12 and D-IK8 increase the uptake and binding of fluorescently labeled vancomycin (bodipy vancomycin). VRSA (VRS10) was incubated with sub-inhibitory concentration of WR12 and D-IK8 for one hour or left untreated. Then treated with bodipy vancomycin for 30 minutes. Bacterial pellets were fixed with 4% paraformaldehyde, and visualized under 40X oil objective of Nikon A1R multi-photon inverted confocal microscope. DIC, differential interference contrast.

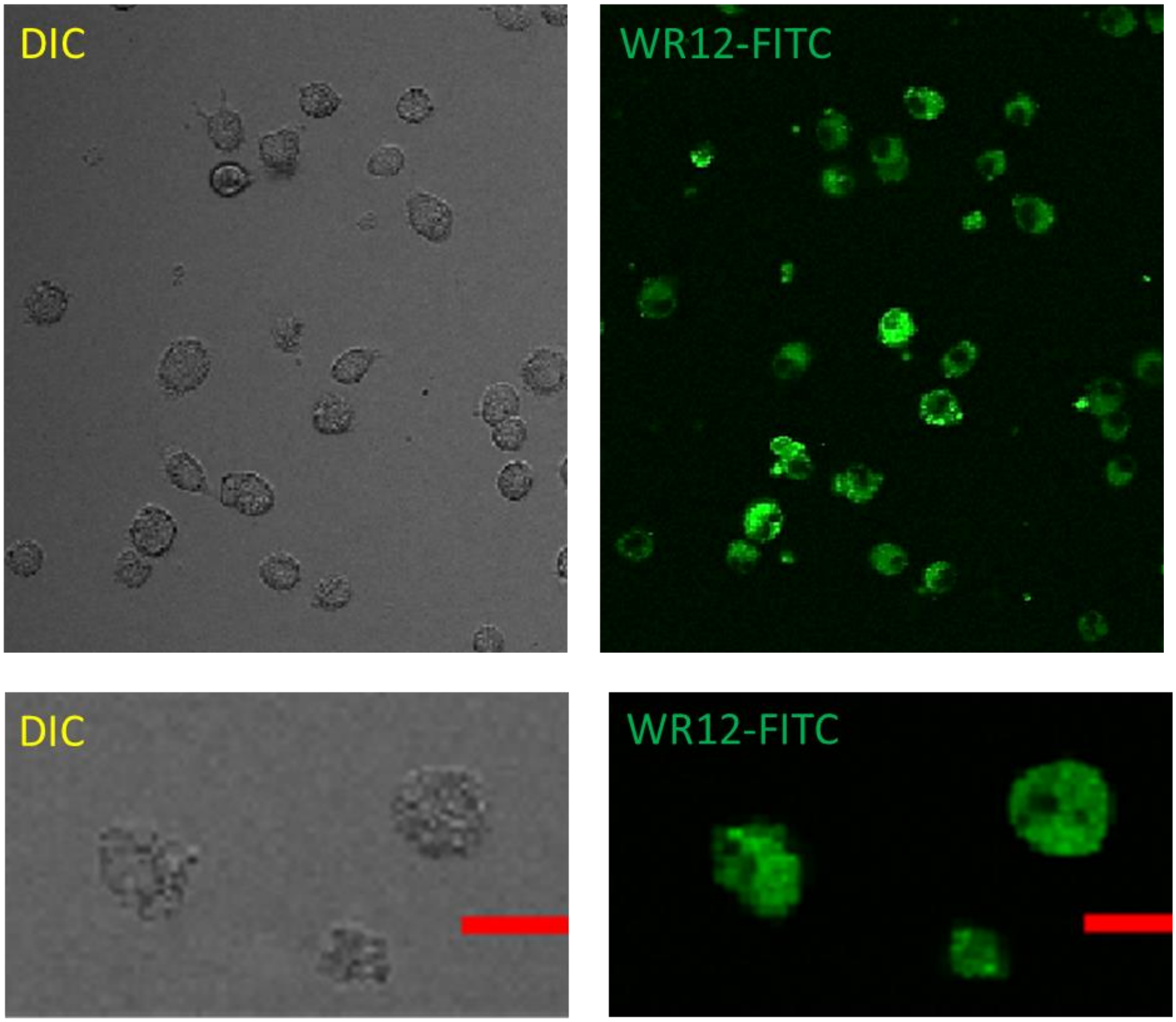

Supplementary figure 4: Internalization studies of WR12: confocal images demonstrate cell penetration of WR12-FITC (10 µM) inside macrophage cells. Lower panel is a higher magnification of upper panel.

Supplementary figures of Mass spectrometry and HPLC of peptides used in the study

1- D-IK8

1.a Mass spectrometry of D-IK8

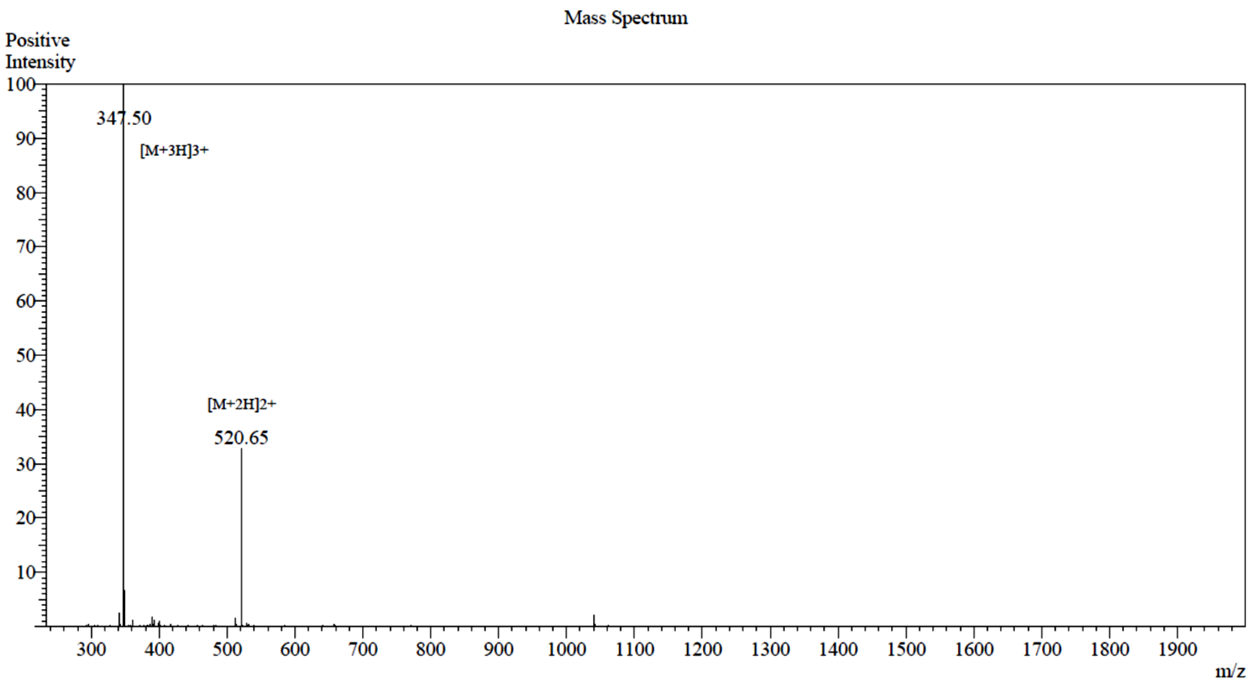

Sample Information	
Acquired by :	Gary
Month-Day Processed :	01/07/14
Time Processed :	17:43:29
Injection Volume :	0.2
Sample Name :	IK8 all D
Sample ID :	372387-7
Theoretical MW :	1039.37
Observed MW :	1039.50

Interface	:ESI
Nebulizing Gas Flow	:1.5L/min
CDL Temp	:250℃
Block Temp	:200℃

Interface Bias	: +4.5 kV
Drying Gas Flow	:5 L/min
T.Flow	:0.2 ml/min
B.conc	:50%H2O/50%MeOH

1.b HPLC of D-IK8

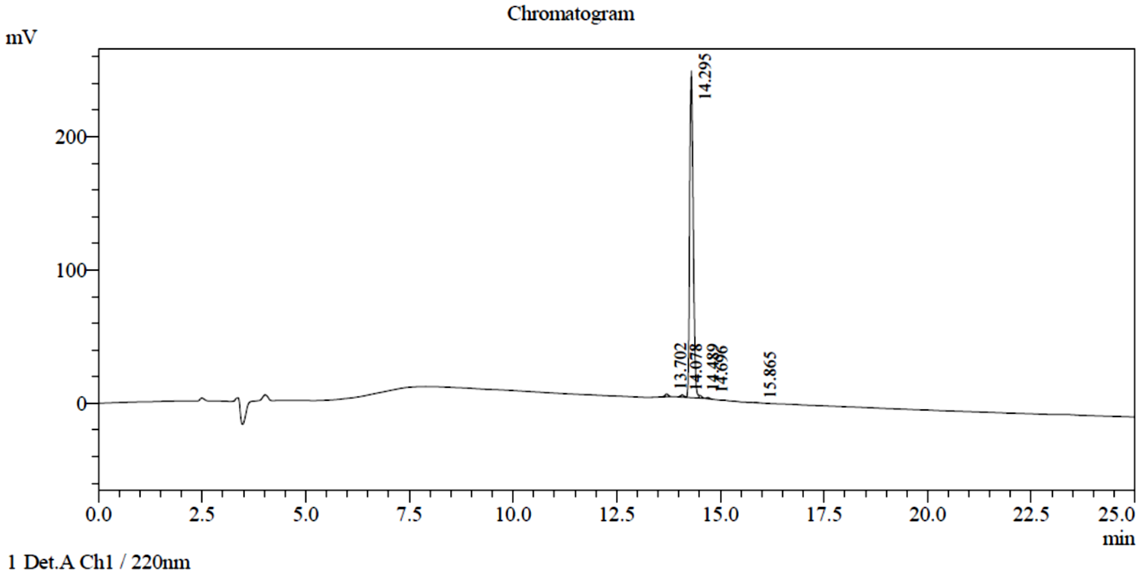

Peak Table

Detector A Ch1 220nm

Peak#	Ret. Time	Area	Height	Area %
1	13.702	13035	2102	0.872
2	14.078	9149	1713	0.612
3	14.295	1456274	245169	97.473
4	14.489	9078	2205	0.608
5	14.696	5005	1039	0.335
6	15.865	1488	258	0.100
Total		1494028	252486	100.000

1- WR12

2.a Mass spectrometry of WR12

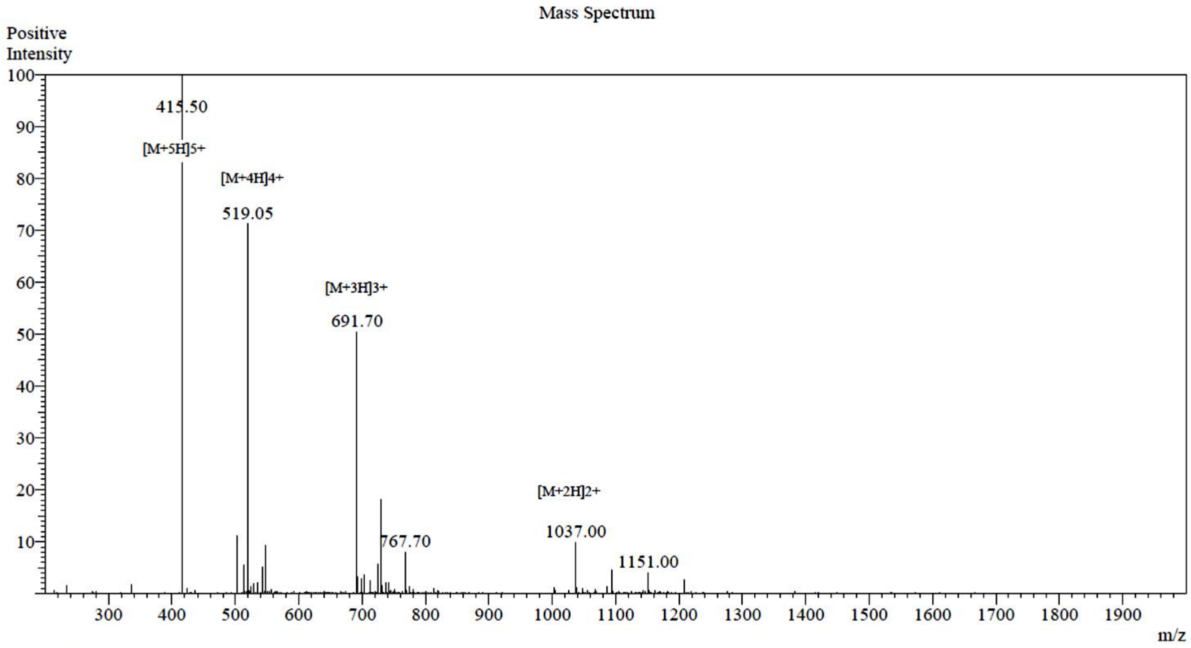

Sample Information
Acquired by : Gary
Month-Day Processed : 09/20/15
Time Processed : 14:58:07
Injection Volume : 0.2
Sample Name : WR-12
Sample ID : 707576-1
Theoretical MW : 2072.40
Observed MW : 2072.50

Interface :ESI
Nebulizing Gas Flow :1.5L/min
CDL Temp :250℃
Block Temp :200℃

Interface Bias : +4.5 kV
Drying Gas Flow :5 L/min
T.Flow :0.2 ml/min
B.conc :50%H2O/50%MeOH

2.b HPLC of WR12

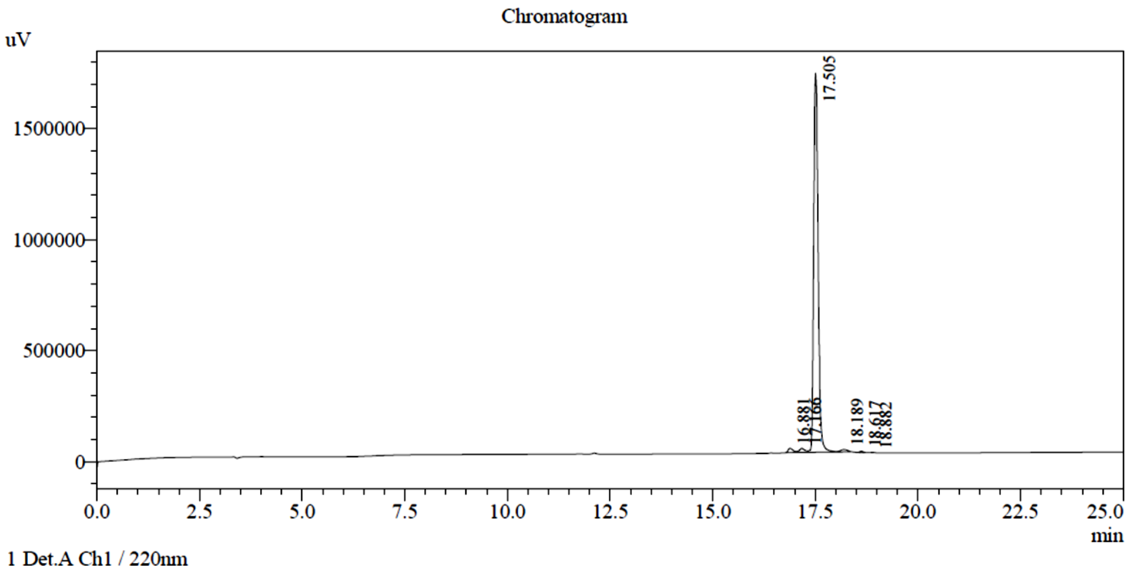

Peak Table

Detector A Ch1 220nm

Peak#	Ret. Time	Area	Height	Area %
1	16.881	161629	19732	1.208
2	17.166	172099	18315	1.286
3	17.505	12862276	1706892	96.144
4	18.189	135864	10680	1.016
5	18.617	36257	6279	0.271
6	18.882	10039	1975	0.075
Total		13378164	1763874	100.000

References

1 **CLSI**. Methods for dilution antimicrobial susceptibility tests for bacteria that grow aerobically; approved standard M7-A7. CLSI, Wayne, PA. (2007).

2 Xiong, Y. Q., Mukhopadhyay, K., Yeaman, M. R., Adler-Moore, J. & Bayer, A. S. Functional interrelationships between cell membrane and cell wall in antimicrobial peptide-mediated killing of Staphylococcus aureus. *Antimicrob Agents Chemother* **49**, 3114-3121, doi:10.1128/AAC.49.8.3114-3121.2005 (2005).

3 Marks, L. R., Clementi, E. A. & Hakansson, A. P. Sensitization of Staphylococcus aureus to methicillin and other antibiotics in vitro and in vivo in the presence of HAMLET. *PloS one* **8**, e63158, doi:10.1371/journal.pone.0063158 (2013).

4 Sakoulas, G. *et al.* Ampicillin enhances daptomycin- and cationic host defense peptide-mediated killing of ampicillin- and vancomycin-resistant Enterococcus faecium. *Antimicrob. Agents Chemother.* **56**, 838-844, doi:10.1128/AAC.05551-11 (2012).
